# Baseline prognostic predictors in classical Hodgkin Lymphoma: a retrospective, single-center analysis on patients treated with PET/CT-guided ABVD

**DOI:** 10.3389/fonc.2024.1419118

**Published:** 2024-09-05

**Authors:** Alessandro Cellini, Chiara Adele Cavarretta, Francesco Angotzi, Valeria Ruocco, Andrea Serafin, Nicolò Danesin, Marco Pizzi, Michele Gregianin, Stefania Vio, Filippo Crimì, Federica Vianello, Francesco Piazza, Livio Trentin, Andrea Visentin

**Affiliations:** ^1^ Hematology Unit, Department of Medicine, University of Padua, Padua, Italy; ^2^ Surgical Pathology and Cytopathology Unit, Department of Medicine, University of Padua, Padua, Italy; ^3^ Nuclear Medicine Unit, Veneto Institute of Oncology IOV-IRCCS, Padua, Italy; ^4^ Radiology Unit, Department of Medicine, University of Padua, Padua, Italy; ^5^ Institute of Radiology, Department of Medicine, University of Padua, Padua, Italy; ^6^ Radiotherapy Unit, Veneto Institute of Oncology IOV-IRCCS, Padua, Italy

**Keywords:** Hodgkin Lymphoma, IPS, NLR, LMR, ABVD

## Abstract

**Introduction:**

The identification of baseline prognostic factors in Classical Hodgkin Lymphoma could help in tailoring a risk-based approach as the therapeutic landscape expands. Currently, the International Prognostic Score (IPS) represents the most used prediction tool in clinical practice, but other potential baseline risk predictors have been identified.

**Methods:**

We performed a retrospective analysis in a cohort of 274 patients treated with ^18^FDG-PET/CT-guided ABVD to assess the prognostic significance of the IPS risk factors, and to validate the impact of the peripheral blood lymphocyte to monocyte (LMR) and neutrophil to lymphocyte (NLR) ratios on prognosis definition.

**Results:**

Among the considered risk factors, stage IV disease (HR 1.83), leukocytosis (HR 2.28), anemia (HR 3.23) and low LMR (HR 2.01) significantly predicted PFS, whereas male sex (HR 2.93), stage IV disease (HR 3.00) and lymphopenia (HR 7.84) significantly predicted OS. A 4 variable and a 3 variable prognostic system was subsequently proposed for PFS and OS, respectively. In both cases, a stark decrease in the survival probability was documented as the score increased. Moreover, by selecting only the significant IPS items and considering a more recently proposed prognostic factor (LMR) we were able to better identify patients at higher risk of relapsing after PET/CT-guided ABVD.

**Discussion:**

Although the IPS was still able to identify a subgroup of high-risk patients within our cohort of individuals treated with PET/CT-guided ABVD, not all the risk factors that it considers were found to have an impact on survival times. Moreover, by selecting only the significant IPS items and considering a more recently proposed prognostic factor (LMR) we were able to better identify patients at higher risk of relapse, in an effort to contribute to the building of a modern risk prediction tool that can help guide treatment choices.

## Introduction

While a definite cure after the first line of therapy is obtained in around 80 to 90% of patients affected by classical Hodgkin Lymphoma (cHL), some still experience disease relapse requiring further lines of therapy, with a significant portion of relapsed and refractory patients (R/R) failing to obtain disease control (1–6). With the advent of novel agents in the therapeutic landscape of cHL (7–9), and their progressive introduction in earlier lines of therapy (10–12), the search for easily measurable and baseline-available prognostic factors becomes of paramount importance to identify patients at a higher risk of relapse or disease-related death, guiding therapeutic strategies to maximize response rates and minimize toxicities and excessive healthcare-associated costs.

To date, the Hasenclever International Prognostic Score (IPS) is the most widely used tool to predict the prognosis of patients affected by cHL (13). Originally proposed in 1998, the score includes a set of 7 dichotomous variables that were found to predict both progression-free survival (PFS) and overall survival (OS). Since then, the introduction of a PET/CT guided approach in frontline therapy and the discovery of novel drugs (namely Brentuximab-Vedotin and checkpoint inhibitors), as well as the improvement of supportive care, have radically changed the therapeutic landscape for cHL (14–16).

In recent years, both lymphocyte-to-monocyte ratio (LMR) and neutrophil-to-lymphocyte ratio (NLR) have been evaluated as potential prognostic factors available at baseline in cHL (17–22). The appeal of such potential prognostic factors resides in them being readily available at baseline after routine analyses, a characteristic they share with the original items included in the IPS. The prognostic efficacy of these factors has been evaluated and validated in different publications, but, to date, they have not been included in a multivariable prognostic model for cHL. We therefore performed a retrospective study in a cohort of patients treated with PET/CT-guided ABVD therapy within the modern era to assess the prognostic value of the single factors initially included in the IPS, as well as to evaluate the contribution of LMR and NLR in prognosis definition.

## Methods

### Patients

Patients diagnosed with cHL at our institution between 2004 and 2022 were included in the analysis. All patients were staged with ^18^F-FDG PET/CT at baseline, with advanced stage cHL being defined as stage IIB or higher. During the staging process, a complete blood count and a biochemistry panel including renal and hepatic function, erythrocyte sedimentation rate (ESR, considered as normal when <30 mm/h), and protein electrophoresis with albumin quantification were performed in all patients. All blood counts were performed through the use of an automated cell counter. Blood film review by laboratory personnel was performed in the case of machine-detected morphological or count abnormalities. In cases where the staging was done elsewhere, the values obtained right before the start of chemotherapy were considered as baseline, excluding patients receiving pre-emptive steroid therapy given its impact on blood counts.

IPS score calculation was performed as previously described, with one point being assigned to each of these variables: male sex, stage IV, age ≥45 years, serum albumin <40 g/L, hemoglobin <105 g/L, white blood cell count (WBC) >15 ✕ 10^9/L or absolute lymphocyte count (ALC) <0.6 ✕ 10^9/L and/or <8% of the WBC.

Response evaluation was performed by means of PET/CT after 2 cycles (interim-PET, iPET) and one month after the end of therapy, with quantification according to the Deauville 5-point scale. PET/CT scans with a Deauville score of 1-3 were considered negative. All PET/CT scans that had areas of uncertainty and all complex cases were thoroughly examined and discussed in multidisciplinary meetings attended by nuclear medicine experts, radiology experts and expert hematologists.

### Therapy

All patients were treated with PET/CT-guided ABVD. Early-stage subjects with a negative iPET scan underwent 2 more ABVD cycles and consolidation radiotherapy. If radiotherapy was not performed due to the anatomic localization of the disease or due to the patient’s or the clinician’s decision, 4 more ABVD cycles were administered instead. Advanced-stage subjects with a negative iPET scan underwent 4 more ABVD cycles.

Patients whose iPET scan was consistent with a partial response (Deauville score of 4) underwent therapy intensification with a shift to escalated-BEACOPP for 2 to 4 cycles in patients having early-stage and advanced-stage cHL, respectively.

Patients with stable disease or progression detected by the iPET scan (Deauville score of 5) were shifted to second-line salvage therapy (IGEV or BeGEV) for 3 to 4 cycles and consolidated with high-dose chemotherapy and autologous stem cell transplantation.

Refractory disease was defined as progression being detected earlier than 3 months from therapy completion.

### Statistical analysis

Baseline continuous risk factors were dichotomized according to the cut-offs specified in the original Hasenclever prognostic score. Low LMR was considered as a ratio lower than 2.1, whereas high NLR was considered as a ratio higher than 6.0, as previously reported (22).

Overall survival (OS) was calculated from the time of the diagnosis to the time of last follow-up or death. Progression-free survival (PFS) was calculated from the time of diagnosis to the time of last follow-up, disease progression or death due to any cause, whichever occurred first. Survival curves were designed according to the Kaplan-Meier method and were compared using the log-rank statistic, and hazard ratios between risk groups were calculated according to the Cox proportional hazards method, with a p of 0.05 considered as the limit for statistical significance. The performance of the prognostic scores was assessed through the means of Harrel’s c-index of concordance.

All calculations were performed using R version 4.2.2 for Windows.

## Results

A total of 302 patients were identified, with 21 being removed due to missing data and 7 being excluded due to having undergone non-PET/CT-guided Brentuximab-AVD.

One hundred and twenty-seven patients (46%) were female, and the median age at diagnosis was 32 years, with 19 patients (7%) being 65 years and older. Early-stage cHL was diagnosed in 108 patients (39%), and 167 patients (61%) had advanced-stage disease. Among them, 57 (21%) had stage IV cHL. Bulky disease was documented in 92 patients (34%), and 135 patients (49%) presented with B symptoms (**Table 1**).

**Table 1:** Characteristics of the patients at baseline.

Characteristic	Patients (n=274)
Male sex - n. (%)	127 (54)
Median age at diagnosis - yr. (IQR; range)	32 (22; 17-83)
Age ≥45 - n. (%)	78 (28)
Age ≥65 - n. (%)	19 (7)
Age ≥75 - n. (%)	3 (1)
Early stage - n. (%)	108 (39)
I - n. (%)	6 (2)
IIA - n. (%)	101 (37)
Advanced stage - n. (%)	167 (61)
IIB - n. (%)	66 (24)
III - n. (%)	44 (16)
IV - n. (%)	57 (21)
B symptoms - n. (%)	135 (49)
Bulky disease - n. (%)	92 (34)
Elevated ESR - n. (%)	205 (75)
ESR ≥50 mm/h - n. (%)	139 (51)
Elevated CRP - n. (%)	204 (74)

CRP, C reactive protein; ESR, Erythrocyte sedimentation rate; IQR, Interquartile range.

A total of 160 patients (58%) had low LMR, and 100 (36%) had high NLR, with 100 (34%) presenting with both risk factors.

### Survival

With a median follow-up time of 63 months (range: 1-198 months; IQR: 74 months), 20 (7%) patients died (of whom 6 had a disease-related cause of death), while 57 (21%) experienced disease progression. Among the latter, 30 (53%) were classified as having a refractory disease. Of note, only one patient had <6 months of follow-up due to an early death during chemotherapy.

The five-year PFS and five-year OS in the whole population were 75.4% (95% CI 69.4-80.3) and 94.3% (95% CI 90.0-96.7), respectively, and the median PFS and OS were not reached.

A significant difference for both PFS (HR 4.75, 95% CI 2.35-9.60; p<0.001) and OS (HR 3.88, 95% CI 1.14-13.26; p<0.05) was documented between early and advanced-stage patients, although neither patient subgroup reached the median PFS or the median OS (**Supplementary Figures S1A, B**).

**Figure 1:**
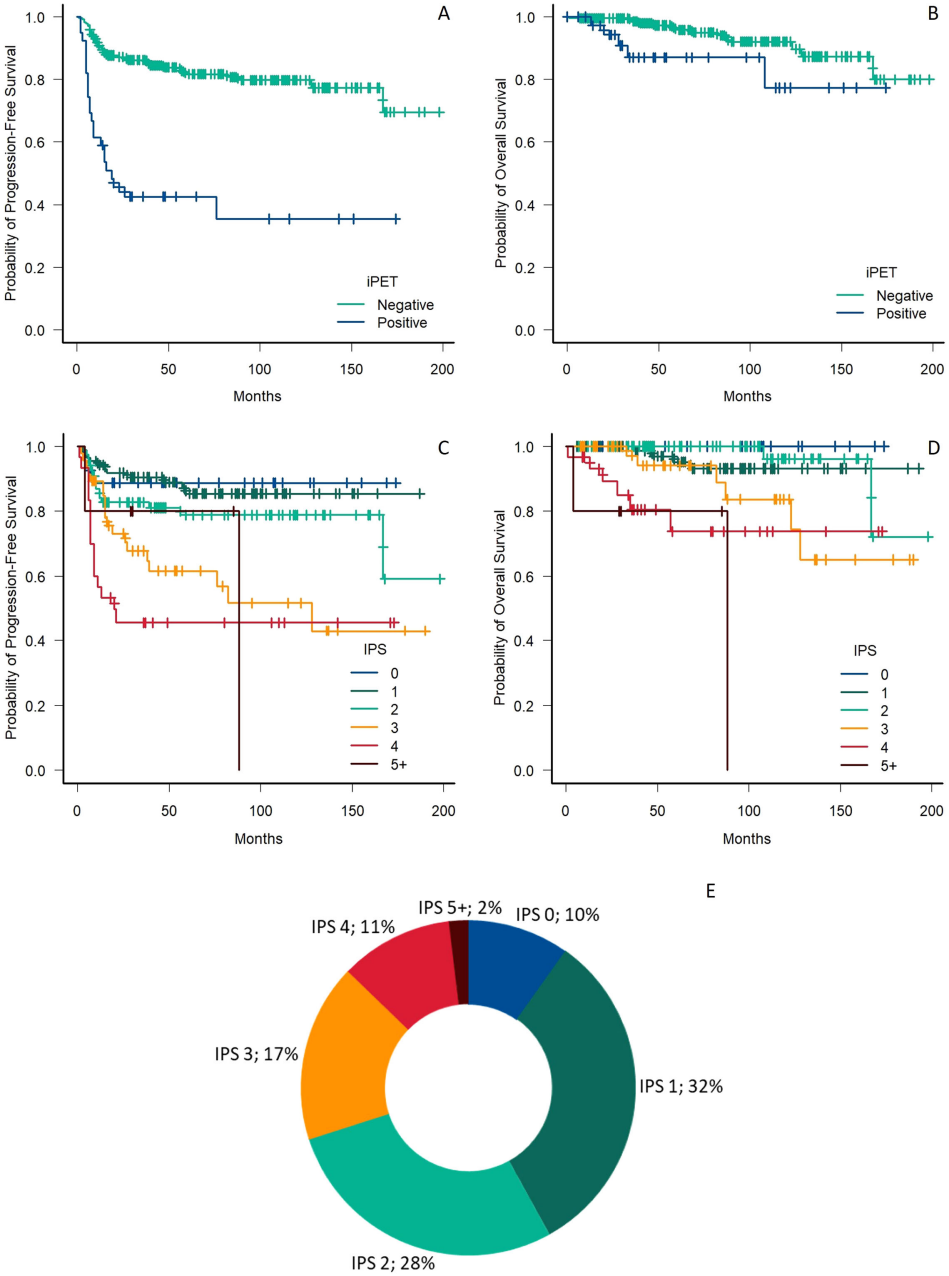
**(A, B)** Comparison of progression-free survival **(A)** and overall survival **(B)** in respect to the iPET status. **(C, D)** Comparison of progression-free survival **(A)** and overall survival **(B)** in respect to the IPS risk group. **(E)** Percentage of patients in each IPS risk group. iPET, interim PET/CT, IPS, International Prognostic Score.

Thirty-nine patients (14%) had a positive interim PET/CT scan, of which 30 (77%) were advanced-stage patients, with 10 patients being iPET positive for each of the advanced stages (IIB, III and IV). Among iPET-positive patients, 4 (10%) shifted to BeGEV or IGEV (3 advanced stage, 1 early stage), whereas the rest were escalated to eBEACOPP. iPET-positive patients had a 5-year PFS and OS of 42.5% (95% CI 26.7-57.5) and 87.0% (95% CI 68.7-95.0), respectively. Both survival times were significantly worse in the iPET-positive patients when compared to the iPET-negative subgroup, with an HR of 4.84 (95% CI 2.90-8.08) for PFS and an HR of 2.90 (95% CI 1.04-8.07) for OS (**Figures 1A, B**).

### IPS-calculated prognosis

Taking into account the original IPS risk factors, 27 (10%) patients had an IPS score of 0, 88 (32%) patients had an IPS score of 1, 77 (28%) patients had an IPS score of 2, 47 (17%) patients had an IPS score of 3, 30 (11%) patients had an IPS score of 4, and 5 patients (2% had an IPS score of 5, with no subjects having a score of 6 or 7 (**Figure 1E**). Although visual inspection of the curves showed a decrease in PFS among risk groups (**Figure 1C**), no statistically significant decrease in 5-year PFS was documented when assessing the difference between adjacent risk groups, with the sole exception of the comparison between subjects having an IPS 3 and those at a lower risk (**Supplementary Table S1**). The same exception held true when comparing OS across the risk groups, with no other significant differences being identified in the comparison between adjacent risk categories (**Figure 1D** and **Supplementary Table S1**). A subsequent analysis was performed after grouping patients having an IPS score of 0 to 2 and 3 or higher, which documented a significant decrease in PFS (p<0.001; HR 3.48, 95% CI 2.14 – 5-36) and OS (p<0.001; HR 6.51, 95% CI 2.50 – 17.00) among the two groups.

### Prognostic factor evaluation

Considering the outcome predictors included in the IPS, as well as LMR and NLR, in univariate analysis, stage IV, low albumin, low hemoglobin, low lymphocyte count, high WBC count, low LMR and high NLR were associated with a worse PFS. In multivariate analysis stage IV, low hemoglobin, high WBC count and low LMR emerged as significant predictors for worse PFS (**Table 2**).

**Table 2:** Evaluation of potential progression-free survival predictors.

Variable	HR (95% CI)	p-value	Multivariate HR	Multivariate p-value
Age	1.00 (0.59 - 1.71)	0.996		
Sex	1.14 (0.71 - 1.84)	0.590		
Stage IV	2.38 (1.45 - 3.93)	<0.001***	1.83 (1.1 - 3.7)	0.021*
WBC count	2.85 (1.62 - 5.01)	<0.001***	2.28 (1.21 - 4.29)	0.011*
Lymphocyte count	2.07 (1.00 - 4.34)	0.049*	1.56 (0.68 - 3.60)	0.297
Hemoglobin	4.60 (2.72 - 7.75)	<0.001***	3.23 (1.83 - 5.68)	<0.001***
Albumin	2.27 (1.23 - 4.01)	0.006**	1.17 (0.61 - 2.24)	0.643
LMR	2.57 (1.44 - 4.43)	<0.001***	2.01 (1.08 - 3.78)	0.028*
NLR	2.00 (1.24 - 3.23)	0.003**	0.837 (0.45 - 1.55)	0.573

NLR, Neutrophil-to-lymphocyte ratio; WBC, White blood cell. *p<0.05; **p<0.01; ***p<0.001.

On the other hand, considering the same potential outcome predictors, older age, male sex, stage IV, low lymphocyte count and low LMR were associated with a lower OS. In multivariate analysis, male sex, stage IV and low lymphocyte count emerged as significant predictors for worse PFS (**Table 3**).

**Table 3:** Evaluation of potential overall survival predictors.

Variable	HR (95% CI)	p-value	Multivariate HR	Multivariate p-value
Age	2.59 (1.07 - 6.30)	0.029*	2.19 (0.85 - 5.62)	0.103
Sex	2.60 (1.00 - 6.77)	0.042*	2.93 (1.07 - 7.98)	0.036*
Stage IV	3.45 (1.43 - 8.34)	0.003**	3.00 (1.13 - 7.93)	0.027*
WBC count	1.45 (0.43 - 4.97)	0.547		
Lymphocyte count	6.93 (2.64 -18.21)	<0.001***	7.84 (2.72 - 22.63)	<0.001***
Hemoglobin	2.38 (0.80 - 7.17)	0.111		
Albumin	1.80 (0.65 - 4.96)	0.249		
LMR	3.10 (1.03 - 9.29)	0.033*	2.116 (0.70 - 6.43)	0.186
NLR	2.29 (0.95 - 5.54)	0.058		

LMR, Lymphocyte-to-monocyte ratio; NLR, Neutrophil-to-lymphocyte ratio; WBC, White blood cell; LMR, Lymphocyte to monocyte ratio. *p<0.05; **p<0.005; ***p<0.001.

We subsequently compared the distribution of the analyzed risk factors among the iPET-positive patients and their iPET-negative counterparts, finding that the proportion of patients having leukocytosis (p<0.05), lymphopenia (p<0.01), low hemoglobin (p<0.05), low LMR (p<0.05) and high NLR (p<0.05) was statistically different among the two groups (**Supplementary Table S3**).

Given such differences in distribution, we performed an exploratory analysis separately evaluating the impact of the considered prognostic factors in patients who ended up being iPET-positive and in those who did not, finding that, in PET2+ patients, no factor impacted PFS and only lymphopenia influenced OS, whereas, in PET2 negative patients, only stage impacted OS and anemia, stage, leukocytosis and low NLR affected PFS, with the latter losing its prediction power in multivariate analysis. The full results of these analyses, along with the HRs for each factor, are presented in **Supplementary Tables S4**–**S6**.

### Modified prognostic score

**Figure 2:**
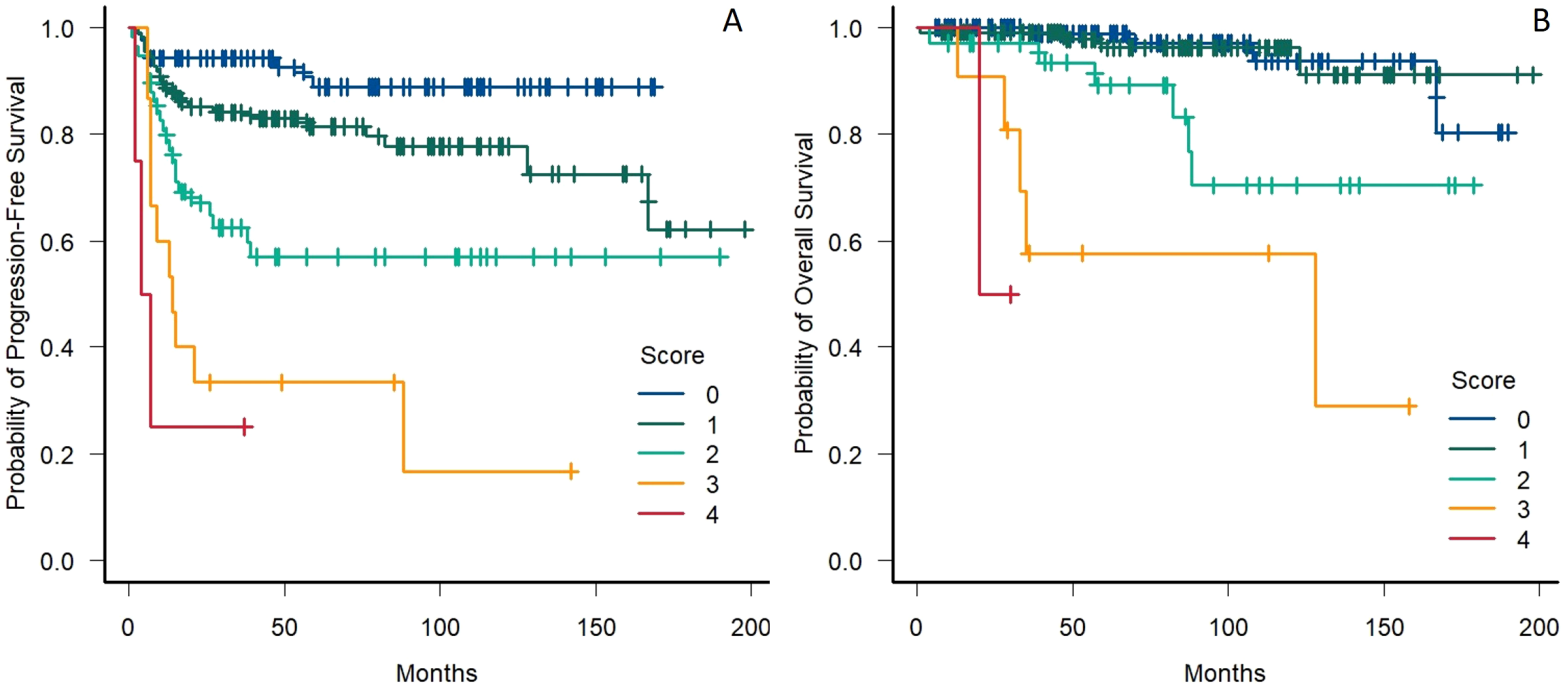
**(A)** Progression-free survival in patients stratified by the presence of stage IV disease, anemia, leukocytosis and low LMR. **(B)** Overall survival in patients stratified by the presence of male sex, stage IV disease and lymphopenia; LMR, Lymphocyte to monocyte ratio.

Based on the relevant factors identified through the multivariate analysis (stage IV, high WBC count, low hemoglobin, low LMR), we then built a modified prognostic score for PFS based on the IPS format, with each of the identified risk factors being attributed a similar weight based on the similarities of their impact on patient prognosis. Thus, in our cohort, 87 (32%) individuals had no risk factors, 110 (40%) had one risk factor, 58 (21%) had two, 15 (5%) had three and 4 (2%) had four. Five-year PFS was significantly different across the five identified risk groups (**Figure 2A**), with a marked drop in PFS probability being documented between patients having 0, 1, 2 and 3 risk factors (HR 2.25, 95% CI 1.78-2.85, across all risk groups). No statistically significant difference in 5-year PFS was identified between the two higher-risk groups (**Supplementary Table S2**). The performance of the proposed prognostic score was subsequently assessed by the means of Harrel’s c-index, yielding a value of 0.71, whereas the IPS yielded a value of 0.68 in the same cohort of patients.

A similar model was then built for the prediction of OS (utilizing male sex, stage IV and low lymphocyte count as risk factors), with double weight being assigned to lymphopenia in comparison with the other risk factors based on the HRs obtained in the multivariate analysis. Thus, 4 risk categories were identified for the prediction of OS, with 114 (42%), 113 (41%), 34 (12%), 11 (4%) and 2 (1%) patients having zero, one, two, three or four points, respectively. A decrease in 5-year OS followed the increase of the risk score (**Figure 2B**), with no difference being documented between subjects having zero or one point, and a statistically significant decrease in the probability of OS being identified in patients having two, three or four points (HR 3.04, 95% CI 1.96-4.72, across all risk groups) (**Supplementary Table S2**). The performance of the proposed prognostic score was comparable to the one of the IPS in the prediction of 5-year OS (c-index 0.79 and 0.77, respectively).

## Discussion

As novel agents are being introduced in the therapeutic landscape for cHL, the identification of patients at a higher risk of relapse or refractoriness after the first line of therapy can help guide treatment choices.

To date, the most widely used prognostic tool for cHL is the Hasenclever IPS, a score built upon a cohort of patients treated with heterogeneous treatment schemes between the 1980s and early 1990s. This score, however, was utilized as an adjuvant in therapeutic decisions only in a handful of clinical trials (5, 23, 24), and is not commonly considered when making treatment choices in our day-to-day clinical practice.

On the other hand, therapy escalation based on iPET results is common in clinical practice, as the lack of an early response, documented by a positive iPET scan, carries a heavy prognostic burden, a finding that was further confirmed by our study. A PET/CT-guided therapy, however, was not able to bridge the gap between our cohort of early responders and their counterparts, and the negative impact of a positive iPET scan carried over to OS, probably owing to a combination of the increased toxicity of the eBEACOPP regimen and the biologically aggressive nature of the disease, which makes the obtainment of an appropriate response hard, even with subsequent salvage therapies.

To further evaluate the predictive power of the IPS, we evaluated survival outcomes after sorting our patients in each IPS risk group, failing to reproduce the stepwise decrease in 5-year PFS across consecutive risk groups described in the original publication. Nevertheless, we identified a significant overlap between different risk groups with the formation of two different clusters, with the higher-risk patients defined as having an IPS ≥3 (with the survival curve depicted by patients with a score ≥5 not being reliable due to them representing only 2% of the sample), a division that was already pointed at in the original manuscript.

Considering these results, we subsequently re-evaluated the prognostic effect of each item initially included in the IPS, finding that, among them, sex and age were not significantly associated with a decrease in PFS. In contrast, WBC count, hemoglobin and albumin levels were not significantly associated with a reduction in OS in our cohort.

Turning our attention to some more recently proposed risk factors, we found that low LMR was associated with a decrease in both OS and PFS, while high NLR was only associated with a decrease in PFS.

Interestingly, only leukocytosis, anemia, low LMR and stage IV disease were found to significantly predict PFS in a multivariate analysis and, when performing a similar analysis on OS predictors, a significant association was identified with lymphopenia, male sex and stage IV.

Having identified a set of variables that held their significance in a multivariate analysis, we then built a 4-factor (stage IV, high WBC count, low hemoglobin, low LMR) and a 3-factor (male sex, stage IV and low lymphocyte count) prognostic score for the prediction of PFS and OS, respectively. The former allowed for clear differentiation between the different risk groups, with patients having ≥2 risk factors harboring a particularly high chance for disease recurrence, with a statistically significant difference in 5-year PFS between the single risk groups, with the sole exception of patients with 3 or 4 risk factors, a finding that can be justified by the very limited number of individuals in the highest category. The proposed prediction tool for OS yielded similar results, barring the statistically significant difference among the two lower risk groups. Both scores performed better than the IPS regarding concordance, although the OS prediction tool had a much narrower margin of improvement.

Given the marked impact carried by iPET positivity on both OS and PFS, we performed exploratory analyses to search for differences in the distribution of the considered baseline risk factors between iPET-positive and iPET-negative subjects, finding significant differences in the proportion of patients having leukocytosis, lymphopenia, low hemoglobin, high NLR and low LMR between the two groups. Furthermore, we separately evaluated the impact on OS and PFS of such risk predictors in the two subgroups of patients, failing to reproduce the findings that we reported in the primary analysis. Taken together, these findings point to the fact that the negative prognostic impact of the evaluated risk factors is at least partially tied to their higher prevalence in patients who end up being iPET-positive. This, however, does not reduce their clinical utility, but rather enhances it, as they allow for the identification of patients at a baseline higher risk of a suboptimal early response to frontline therapy, which is considered to be one of the strongest negative prognostic indicators in cHL.

On balance, while it can achieve a relatively high cure rate, our current PET/CT-guided clinical practice for cHL is still unsatisfactory, as a relevant portion of patients does not achieve a cure after first-line therapy, and we are not able to nullify the adverse impact carried by iPET positivity both on overall- and disease-free survival. The development of prognostic models that can help make risk-adapted treatment choices is an essential milestone in the journey towards the cure for cHL, and the currently available tools are incomplete in this regard, either by having been developed in another era and thus not being applicable to current patients, or by not allowing for the identification of risk groups (25). It is interesting to note that the factors which significantly correlated with worse PFS in our analysis were more representative of the disease’s activity and the systemic inflammation often associated with HL, with none of them, with the sole exception of lymphopenia, being significantly associated with OS. This could indicate that currently available salvage strategies (be it high-dose chemotherapy and autologous stem cell transplantation or novel drugs) can effectively nullify these factors’ negative impact. After proper confirmation, this finding could be woven into the design of future clinical trials to identify a cohort of patients that would benefit the most from the upfront utilization of novel therapies.

Our study was mainly limited by its retrospective and monocentric nature which allowed for the enrolment of a limited number of patients across quite a lengthy observation time. Moreover, the study analyzed patients undergoing induction with PET/CT-guided ABVD, whereas this therapeutic paradigm is expected to shift both because of the introduction of BV in the first line of therapy (11) and because of the preliminary results of a number undergoing clinical trials. However, none of these treatment plans contemplates a risk-adapted approach based on baseline factors, with some of them utilizing a different set of characteristics to identify early unfavorable cHL as enrollment criteria (10, 26). Nonetheless, our study provides further evidence on the ability of some baseline patient characteristics to predict the prognosis of cHL patients treated within the modern era. Of note, the factors analyzed in this study can be obtained after history-taking and routine blood work, making them appealing to clinicians treating cHL patients in any area of the world.

We think that our work will contribute to the building of newer risk prediction models for this disease that could be fully integrated into our decision-making, to increase the already high cure rates of cHL and to avoid unnecessary toxicities.

## Data Availability

The raw data supporting the conclusions of this article will be made available by the authors upon reasonable request, without undue reservation.

## Supplementary material

Supplementary Figure 1
**(A, B)** Comparison of progression-free survival **(A)** and overall survival **(B)** in respect to the disease stage (Early stage: I to IIA; Advanced stage: IIB to IV).
