# Supplementary figures and images for: Baseline prognostic predictors in classical Hodgkin Lymphoma: a retrospective, single-center analysis on patients treated with PET/CT-guided ABVD
Source: Front Oncol. 2024 Sep 5;14:1419118. doi: 10.3389/fonc.2024.1419118 (PMC11410762; doi:10.3389/fonc.2024.1419118)

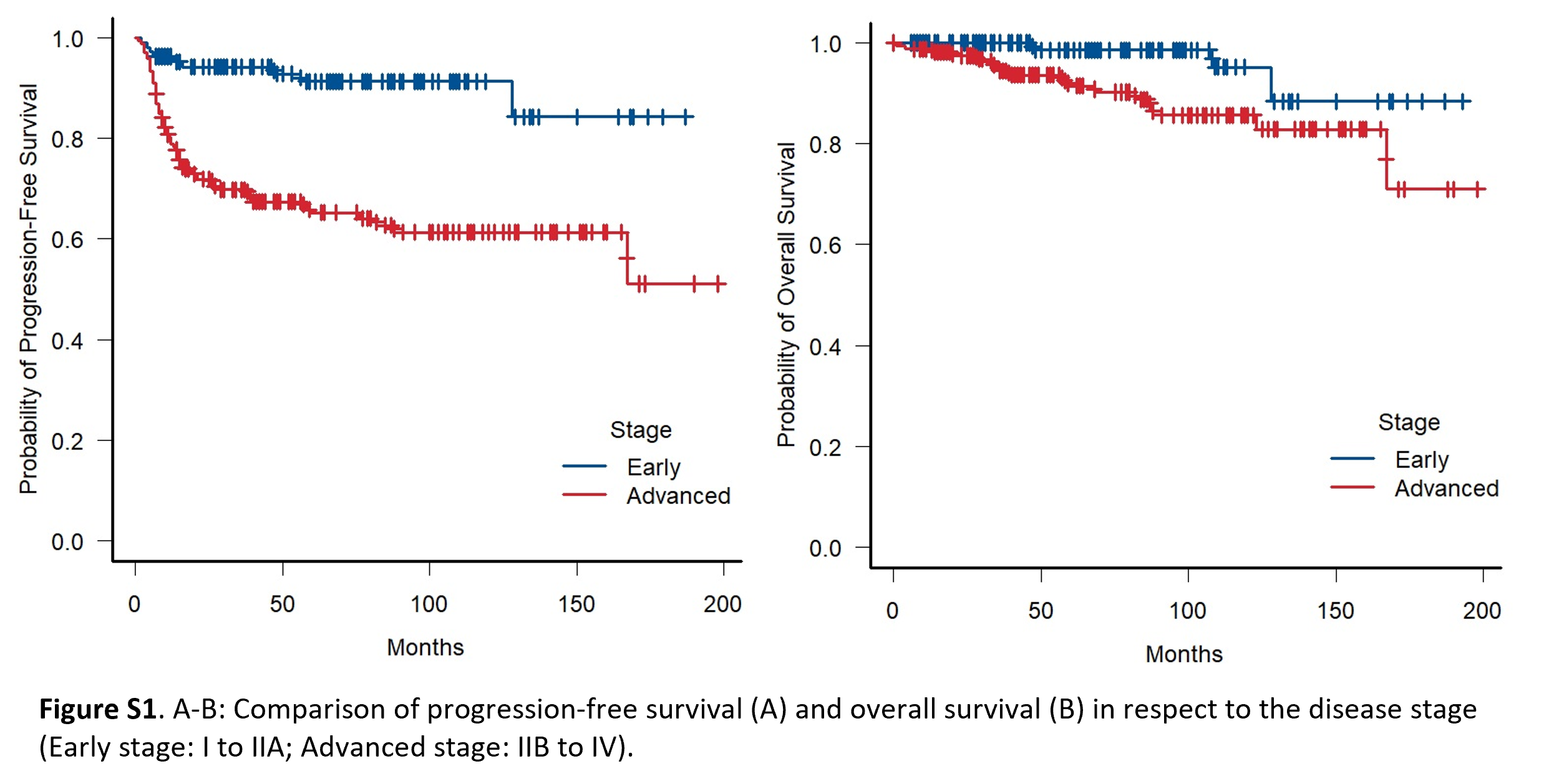

Supplement: Supplementary Figure 1 — (A, B) Comparison of progression-free survival (A) and overall survival (B) in respect to the disease stage (Early stage: I to IIA; Advanced stage: IIB to IV). [file Image1.png]
