# Supplementary material for: Baseline prognostic predictors in classical Hodgkin Lymphoma: a retrospective, single-center analysis on patients treated with PET/CT-guided ABVD
Source: Front Oncol. 2024 Sep 5;14:1419118. doi: 10.3389/fonc.2024.1419118 (PMC11410762; doi:10.3389/fonc.2024.1419118)
Supplement: Supplementary file 2 [file Table1.docx]

**Supplementary tables**

**Table S1**. Comparison of PFS and OS between adjacent IPS risk groups. *p<0.05. HR: hazard ratio; IPS: international prognostic score; OS: overall survival; PFS: progression-free survival.

| **Comparison** | **PFS** | | **OS** | |
| --- | --- | --- | --- | --- |
|  | p-value | HR | p-value | HR |
| IPS 0 vs IPS 1 | 0.92 | 1.07 (0.30-3.84) | 1 | N/A |
| IPS 1 vs IPS 2 | 0.17 | 1.72 (0.80-3.74) | 0.39 | 0.47 (0.08-2.63) |
| IPS 2 vs IPS 3 | 0.02* | 2.14 (1.1-4.17) | 0.04* | 5.29 (1.10-26.28) |
| IPS 3 vs IPS 4 | 0.15 | 1.64 (0.84-3.19) | 0.23 | 2.00 (0.64-6.24) |
| IPS 4 vs IPS 5+ | 0.60 | 0.67 (0.15-2.93) | 0.35 | 2.13 (0.43-10.60) |

**Table S2**. Comparison of PFS and OS between adjacent risk groups as defined by the presence or absence of risk factors found to be significant in multivariate analysis for PFS and OS. *p<0.05; **p<0.01 HR: hazard ratio; IPS: international prognostic score; OS: overall survival; PFS: progression-free survival.

| **Comparison** | **PFS** | | **OS** | |
| --- | --- | --- | --- | --- |
|  | p-value | HR | p-value | HR |
| Score 0 vs 1 | 0.04* | 2.31 (1.03-5.17) | 0.91 | 1.1 (0.27-4.40) |
| Score 1 vs 2 | 0.008** | 2.22 (1.24-4.00) | 0.015* | 4.83 (1.36-17.12) |
| Score 2 vs 3 | 0.02* | 2.36 (1.14-4.89) | 0.04* | 3.45 (1.04-11.45 |
| Score 3 vs 4 | 0.3 | 1.98 (0.54-7.27) | 0.36 | 3.10 (0.28-34.79) |

**Table S3**. Distribution of the potential risk predictors in the iPET positive and iPET negative patients.

| **Variable** | **iPET negative** | **iPET positive** | **p-value** | **OR (95% CI)** |
| --- | --- | --- | --- | --- |
| Age | 66 (28%) | 9 (23%) | 0.566 | - |
| Sex | 109 (47%) | 17 (43%) | 0.732 | - |
| Stage | 46 (20%) | 10 (26%) | 0.397 | - |
| WBC count | 25 (11%) | 10 (26%) | 0.017* | 2.85 (1.11 - 6.95) |
| Lymphocyte count | 11 (5%) | 7 (18%) | 0.007** | 4.38 (1.34 - 13.46) |
| Hemoglobin | 23 (10%) | 9 (23%) | 0.029* | 2.73 (1.01 - 6.85) |
| Albumin | 148 (63%) | 31 (79%) | 0.067 | - |
| LMR | 128 (55%) | 30 (77%) | 0.013* | 2.72 (1.20 - 6.83) |
| NLR | 78 (33%) | 20 (51%) | 0.046* | 2.09 (1.01 - 4.40) |

iPET: Interim PET; LMR: Lymphocyte-to-monocyte ratio; NLR: Neutrophil-to-lymphocyte ratio; WBC: White blood cell. *p<0.05; **p<0.005; ***p<0.001.

**Table S4**. Differential evaluation of potential progression-free survival predictors in iPET negative and iPET positive patients

| **Variable** | **iPET negative** | | **iPET positive** | |
| --- | --- | --- | --- | --- |
|  | **HR (95% CI)** | **p-value** | **HR (95% CI)** | **p-value** |
| Age | 0.95 (0.48 – 1.87) | 0.884 | 1.31 (0.51 – 3.33) | 0.564 |
| Sex | 1.19 (0.65 – 2.18) | 0.569 | 1.06 (0.47 – 2.41) | 0.887 |
| Stage | 3.10 (1.67 – 5.74) | 0.0001*** | 1.05 (0.41 – 2.70) | 0.909 |
| WBC count | 2.93 (1.40 – 6.14) | 0.003** | 1.29 (0.51 – 3.29) | 0.585 |
| Lymphocyte count | 0.97 (0.23 – 4.02) | 0.967 | 1.23 (0.46 – 3.32) | 0.675 |
| Hemoglobin | 4.93 (2.51 – 9.70) | <0.0001*** | 1.76 (0.72 – 4.34) | 0.201 |
| Albumin | 1.55 (0.79 – 3.03) | 0.195 | 3.7 (0.86 – 15.84) | 0.54 |
| LMR | 2.17 (1.11 – 4.24) | 0.02* | 1.64 (0.55 – 4.82) | 0.359 |
| NLR | 1.67 (0.91 – 3.06) | 0.1 | 1.64 (0.71 – 3.77) | 0.235 |

iPET: interim-PET; NLR: Neutrophil-to-lymphocyte ratio; WBC: White blood cell. *p<0.05; **p<0.01; ***p<0.001.

**Table S5**. Results of a multivariate analysis taking into account only risk factors found to be significant predictors of PFS in univariate analysis in the iPET negative patient subgroup

| **Variable** | **HR (95% CI)** | **p-value** |
| --- | --- | --- |
| Stage | 2.42 (1.29 – 4.55) | 0.006** |
| WBC count | 2.16 (1.01 – 4.61) | 0.047* |
| Hemoglobin | 3.37 (1.68 – 6.78) | <0.001*** |
| LMR | 1.67 (0.83 – 3.31) | 0.144 |

iPET: interim-PET; NLR: Neutrophil-to-lymphocyte ratio; WBC: White blood cell. *p<0.05; **p<0.01; ***p<0.001.

**Table S6**. Differential evaluation of potential overall survival predictors in iPET negative and iPET positive patients. HRs were not evaluable for albumin and WBC count in the iPET positive subgroup due to the lack of events in the iPET-positive patients not having the risk factor.

| **Variable** | **iPET negative** | | **iPET positive** | |
| --- | --- | --- | --- | --- |
|  | **HR (95% CI)** | **p-value** | **HR (95% CI)** | **p-value** |
| Age | 2.39 (0.83 – 6.90) | 0.097 | 2.12 (0.35 – 12.78) | 0.402 |
| Sex | 2.59 (0.81 – 8.28) | 0.095 | 1.97 (0.33 – 11.80) | 0.452 |
| Stage | 3.24 (1.16 – 9.36) | 0.021* | 2.56 (0.41 – 15.74) | 0.299 |
| WBC count | 1.59 (0.35 – 7.12) | 0.541 | NE | 0.199 |
| Lymphocyte count | 3.36 (0.74 – 15.22) | 0.095 | 27.52 (3.00 – 252.7) | <0.001*** |
| Hemoglobin | 1.91 (0.42 – 8.62) | 0.393 | 1.01 (0.11 – 9.20) | 0.994 |
| Albumin | 1.18 (0.39 – 3.54) | 0.770 | NE | 0.241 |
| LMR | 2.98 (0.83 – 10.71) | 0.079 | 1.81 (0.20 – 16.38) | 0.593 |
| NLR | 1.40 (0.48 – 4.07) | 0.531 | 5.12 (0.56 – 46.96) | 0.11 |

iPET: interim-PET; NE: not evaluable; NLR: Neutrophil-to-lymphocyte ratio; WBC: White blood cell. *p<0.05; **p<0.01; ***p<0.001.
